# The causality of physical activity status and intelligence: A bidirectional Mendelian randomization study

**DOI:** 10.1371/journal.pone.0289252

**Published:** 2023-08-01

**Authors:** Zhangmeng Xu, Furong Zhang, Guorong Qiu, Yushan Shi, Duoduo Yu, Guogang Dai, Tianmin Zhu

**Affiliations:** 1 Department of Health Preservation and Rehabilitation, Chengdu University of Traditional Chinese Medicine, Chengdu, Sichuan, China; 2 Department of Physical Education, Chongqing University of Arts and Sciences, Chongqing, China; 3 Department of Medical Laboratory, The Affiliated Hospital of Shandong University of Traditional Chinese Medicine, Jinan, Shandong, China; 4 Department-2 of Neck Shoulder Back and Leg Pain, Sichuan Province Orthopedic Hospital, Chengdu, Sichuan, China; The First Hospital of Jilin University, CHINA

## Abstract

**Background:**

Observational studies suggest physical activity (PA) enhances intelligence, while sedentary behavior (SB) poses a risk. However, causality remains unclear.

**Methods:**

We extracted genetic instruments from large genome-wide association studies summary data and employed an inverse-variance weighted (IVW) approach within a random-effects model as the primary method of Mendelian randomization (MR) analysis to estimate the overall effect of various physical activity statuses on intelligence. To assess IVW stability and MR sensitivity, we also utilized supplementary methods including weighted median, MR-Egger, and MR-PRESSO. Furthermore, multivariable MR analysis was conducted to examine the independent effects of each physical activity trait on intelligence.

**Results:**

The MR primary results indicated that LST was negatively associated with intelligence (β = -0.133, 95%CI: -0.177 to -0.090, p = 1.34×10–9), while SBW (β = 0.261, 95% CI: 0.059 to 0.463, p = 0.011) may have a positive effect on intelligence; however, MVPA and SC did not show significant effects on intelligence. Inverse causality analyses demonstrated intelligence significantly influenced all physical activity states.

**Conclusions:**

Our study highlights a bidirectional causal relationship between physical activity states and intelligence.

## Introduction

Intelligence is described as an ability related to a level of cognition that includes the ability to plan, reason, solve problems, think abstractly, learn from experience and understand complex ideas [1]. It reflects the broader and deeper ability to understand our surroundings, which is a very common mental cognitive ability and can be assessed by using a variety of neurocognitive tests [2, 3]. Researches have shown that intelligence is closely linked to heredity [4], and influenced by other environmental factors [5]. Physical activity (PA) was defined by the WHO as any bodily movement produced by skeletal muscles that requires energy expenditure [6]. Strong evidence supports that regular PA has a wide range of health benefits, such as reducing the risk of cardiovascular disease [7], type 2 diabetes [8], and delaying age-related neurological deterioration and cognitive impairment [9], whereas sedentary behavior (SB) may have a negative impact on physical and cognitive function [10, 11].

Intelligence is affected by PA has been a growing concern in recent years. Studies have shown that PA and sedentary time can alter intelligence by affecting subcortical brain structures [12]. Similarly, higher levels of PA during pregnancy were associated with a lower risk of low intelligence scores in early adulthood in offspring [13]. Such studies have made good attempts to explore the correlation between PA and intelligence. However, there are still several questions to be answered. Firstly, does PA affect intelligence with a reverse causality? For instance, among many individuals with cerebral palsy, both their intellectual and cognitive function are in disorder, and the ability to participate in PA is significantly lower than the normal [14]. Infants with Down syndrome learn to walk about one year later than those nondisabled [15]. Secondly, when potential confounders are minimized, does the causal relationship between PA and intelligence still exist? Observational studies inherently cannot eliminate potential confounders, which inevitably has some impact on the findings. Angevaren et al. [16] reported that aerobic PA was beneficial to cognitive function in healthy elder adults, while the opposite conclusion was presented in Greene’s study [17]. Since the relationship between sedentary and cognitive levels is being heatedly debated [18, 19], the potential causality between PA and intelligence also faces such a dilemma.

**Fig 1:**
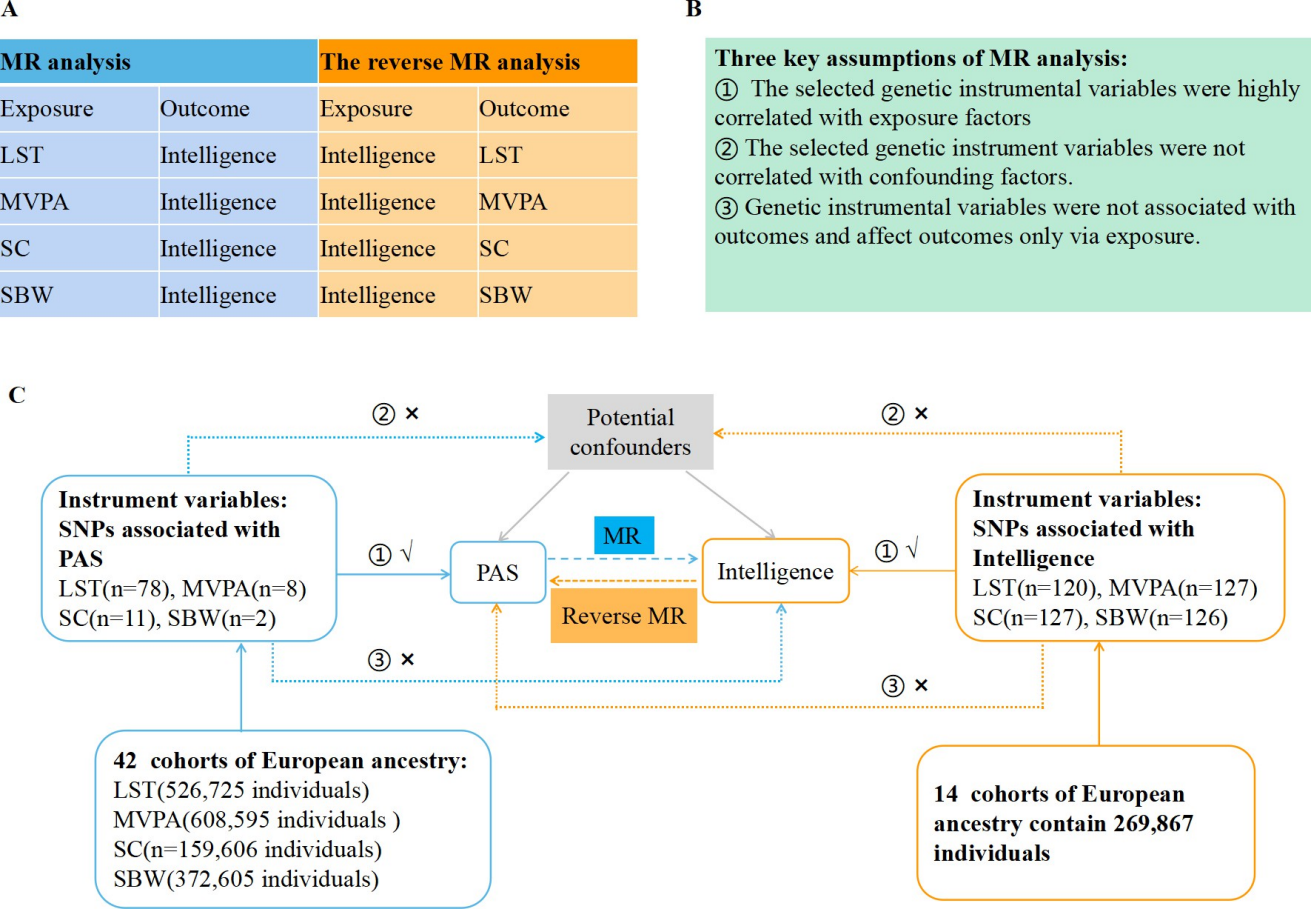
Overview of the study design in this bidirectional MR study. **A.** Eight MR analysis to investigate the bidirectional association between PAS and intelligence. **B.** The three key assumptions of MR analysis. **C.** Sketch of the study design. MR: Mendelian randomization. PAS: Physical activity status; LST: Leisure screen time; MVPA: Moderate-to-vigorous intensity physical activity during leisure time; SC: Sedentary commuting; SBW: Sedentary behavior at work; SNP: Single nucleotide polymorphism.

Mendelian randomization (MR) study is a novel tool for genetic analysis based on the principle that genetic alleles are naturally randomly distributed in a population. It aims to investigate whether there is a causal association between exposure factors and outcome events by analyzing genetic variants [20]. Since genetic variants are determined before embryo formation, they are free from errors caused by acquired diseases and other confounding factors. The MR study is a reliable way for causality extrapolation, which can overcome the limitations of observational studies [21, 22]. In this study, we used a bidirectional two-sample MR analysis to examine the potential causal relationships between different physical activity states (PAS) and intelligence. (Fig 1).

## Methods

### Study design

This study is based on the open genome-wide association studies (GWAS) database, with all original studies included were of ethical approvals. To investigate the causal effect of different PAS on intelligence, we selected moderate-to-vigorous intensity physical activity during leisure time (MVPA), leisure screen time (LST), sedentary commuting (SC), and sedentary behavior at work (SBW) as PAS exposures. In the reverse MR analysis, we assessed the causal relationship of intelligence to each PAS. MR analysis, which used instrumental variables (IV) to infer causal relationships between exposure factors and outcomes, requires to meet the following three key assumptions: 1) The selected genetic IVs are highly correlated with exposures. 2) The selected genetic IVs are not associated with any confounders. 3) Genetic IVs are independent of the outcome and affect outcomes only via exposure (Fig 1B).

### Data sources for PA

We used the largest published PA-related GWAS summarized database, which included data from up to 703,901 individuals from 51 studies [23]. The PA data of the participants were obtained via self-report, LST is a continuous variable defined as the time spent watching TV (hours/day). MVPA is defined as engaging in recreational/vigorous activity greater than or equal to 3MET for more than 1 hour per week. SC is defined as always driving or taking public transportation to and from work. SBW is defined as both at home and at work that mostly requires sitting and only lightly physically intensity or less. To minimize the effect of ethnic disparities on IV, we extracted all the data of the European population (661,399 participants of European ancestry) for MR analysis (S1 Table in S2 File).

### Data sources for intelligence

Intelligence-related data was derived from the study by Savage JE et al. [3], which included GWAS summary data from 14 cohorts of 269,867 individuals of European ancestry (S2 Table in S2 File). Intelligence is a continuous variable, as demonstrated by cognitive test scores.

### Selection of genetic instruments

**Fig 2:**
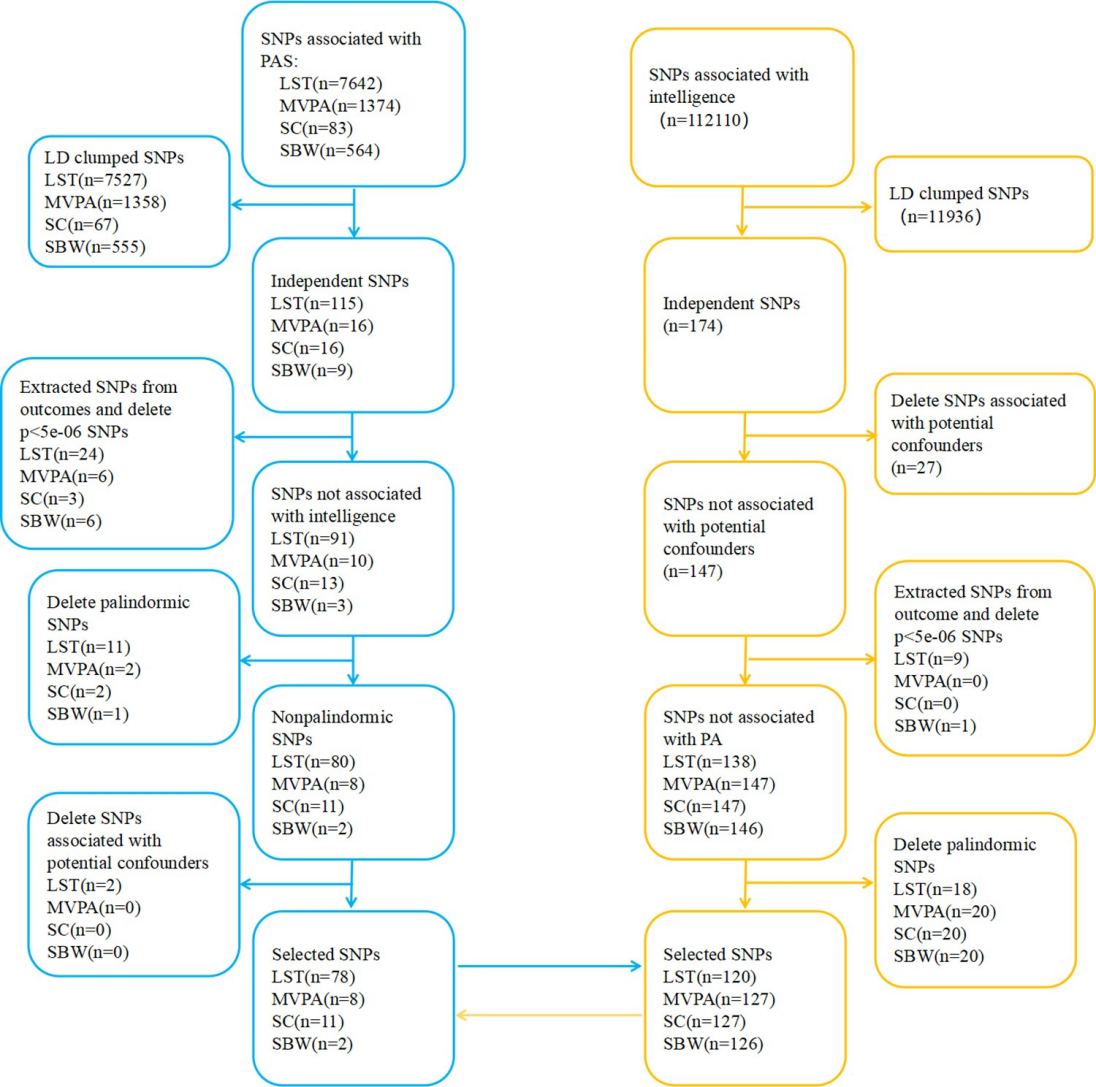
Flow chart of SNPs screening. SNP: Single nucleotide polymorphism; PAS: Physical activity status; LST: Leisure screen time; MVPA: Moderate-to-vigorous intensity physical activity during leisure time; SC: Sedentary commuting; SBW: Sedentary behavior at work; LD: Linkage disequilibrium.

A stepwise screening of IV SNPs for each exposure was performed according to the three MR assumptions (Fig 2). First, we extracted SNPs significantly associated with exposure (*p<*5×10^−8^) from the GWAS database, (*p<*5×10^−6^ due to insufficient SNPs in the SC-related GWAS database), and then we set the screened SNPs independently based on a window of 10 Mb and a threshold of r2<0.001 (Assumption 1). After extracting exposure-associated SNPs from the outcomes, we removed SNPs linked to the outcomes based on a threshold of *p<*5×10^−6^ (Assumption 3). In cases where the IV cannot be matched in the outcomes summary data, high linkage disequilibrium (r2>0.8) proxy SNPs can be identified using https://ldlink.nci.nih.gov/. [24]. Finally, we removed palindromic SNPs and those associated with potential confounders (Assumption 2). The F-test statistic was calculated to quantify the strength of IV, with a threshold of F >10 for MR analyses.

### Statistical analyses

Inverse variance weighting (IVW) was used as the primary method to estimate potential bidirectional causality between different PAS and intelligence, for it can provide reliable estimates of causality in the absence of directional pleiotropy. In addition, we performed complementary analysis using the weighted median and MR-Egger methods because they provide more robust estimates across a wider range of scenarios, albeit with lower efficiency (wider CIs). All analyses were performed using a random effect model. Due to the scored values of intelligence are continuous variables, we used β and 95% confidence intervals (CI) presented the results. Conversely, PAS uses the odds ratio (OR) and 95% CI to demonstrate. Then, we tested for horizontal pleiotropy by MR-Egger intercept. We also tested the heterogeneity of MR-Egger regression and IVW methods by Cochran’s Q-statistic and funnel plots. Additionally, sensitivity analysis was performed using the leave-one-out method. As part of the sensitivity analysis, we present the results of adding proxy SNPs in a supplementary table. We performed eight MR analyses, with statistical significances determined by Bonferroni correction: *p<*0.05/8 (6.25×10^−3^). The *p*-value less than 0.05 but higher than 6.25×10^−3^ was regarded of potential association, which requires further confirmation. The significance level for the MR-Egger and heterogeneity tests was set to *p*<0.05. All *p*-values were two-tailed. Moreover, we used MR-PRESSO to determine the stability of the results and the heterogeneity test.

We noticed that when multiple associated risk factors are used to simultaneously estimate the causal effects of each risk factor on the outcome, this “multivariable Mendelian randomization” approach is similar to the simultaneous assessment of several treatments in a factorial randomized trial [25], where the correlation between risk factors may have an impact on the outcome, so we also used multivariable MR analysis [26] to investigate the independent effects of each PAS on intelligence when PA was used as an exposure factor, the results are intended as an exploratory study only.

## Results

### Instrumental variables SNPs

For SNPs selection, we strictly adhered to the pre-defined protocol (Fig 2). When PA was used as the exposures, LST (n = 78), MVPA (n = 8), SC (n = 11), and SBW (n = 2) were applied as IV (S3-S6 Tables in S2 File). In the reverse direction, 147 SNPs were initially screened, and after merging with PA GWAS data, LST (n = 120), MVPA (n = 127), SC (n = 127), and SBW (n = 126) SNPs were selected as IVs for investigating the causal relationship of intelligence on PA (S7-S10 Tables in S2 File). The results of the search for proxy SNPs are shown in the supplementary table (S13 Table in S2 File). During the screening process, we excluded SNPs related to potential confounders. After reviewing the documents, we concluded that schizophrenia [27], stress [28], obesity and height-based body mass index [29, 30] may be potential confounders. Therefore, we used PhenoScanner (www. phenoscanner.medschl.cam.ac.uk), a platform for comprehensive information on genotypic and phenotypic associations, to determine whether IV SNPs are associated with potential confounders. Besides, we calculated the F-statistic for each SNP, with all meeting F-statistic >10 (S3-S10 Tables in S2 File).

### The causal effect of PAS on intelligence

The results of the MR analysis are presented in Table 1. LST was a risk factor for intelligence (β = -0.133, 95% CI: -0.177, -0.090, *p* = 1.34×10^−9^), which was consistent with the results of the weighted median model. In addition, the result revealed no horizontal pleiotropy (Intercept = -0.002, *p =* 0.419) (S1 Fig in S1 File). To explore the effect of heterogeneity, we performed a leave-one-out test, which showed no significant change in results after removing any individual SNP (S2 Fig in S1 File). In addition, we mapped funnel plots, and no significant asymmetry was found, either (S3 Fig in S1 File). MR-PRESSO analysis showed the causal relationship of LST reducing intelligence remained after removing the outliers (S11 Table in S2 File). Therefore, we believe that the results are reliable. However, for the causality analysis of the other PAS on intelligence, SBW (β = 0.261, 95% CI: 0.059 to 0.463, *p =* 0.011) may promote intelligence, while MVPA (β = -0.007, 95% CI: -0.131 to 0.117, *p =* 0.908) and SC (β = 0.001, 95% CI: -0.087 to 0.088, *p =* 0.989) have no significant effects on intelligence. The MR-Egger and weighted median analyses were consistent with IVW. However, the IV for SBW only had 2 SNPs, which could not be analyzed by MR-Egger and weighted median, so we showed the results for IVW only. We still performed sensitivity analyses for negative results, and the specific results were displayed in the S1-S4 Figs in S1 File. The results of the analysis that include proxy SNPs are consistent with the primary findings. (S14 Table in S2 File).

**Table 1:** Results of Mendelian randomization analysis of PAS on intelligence.

Direction (No.SNPs, *F-statistic*)	Methods	Beta (95%CI)	Beta *P-*value	Cochran’s Q *P*-value	MR-Egger intercept	Intercept *P*-value	MR-PRESSO *P-*value
LST→Intelligence (n = 78, *F* = 140.68)	MR-Egger	-0.055 (-0.249, 0.138)	0.576	1.59×10^−12^	-0.002	0.419	0.984
	IVW	-0.133(-0.177, -0.090)	1.34×10^−9^				
	Weighted median	-0.096 (-0.142, -0.050)	4.16×10^−5^				
	multivariable MR	-0.094 (-0.146, -0.043)	3.35×10^−4^				
MVPA→Intelligence (n = 8, *F* = 187.00)	MR-Egger	-0.350 (-0.944, 0.243)	0.291	0.012	0.009	0.291	NA
	IVW	-0.007 (-0.131, 0.117)	0.908				
	Weighted median	-0.092 ( -0.209, 0.025)	0.121				
	multivariable MR	0.028 (-0.073, 0.129)	0.587				
SC→Intelligence (n = 11, *F* = 112.53)	MR-Egger	0.163 (-0.036, 0.362)	0.144	6.48×10^−6^	0.006	0.115	0.688
	IVW	0.001 (-0.087, 0.088)	0.989				
	Weighted median	0.006 (-0.060, 0.072)	0.854				
	multivariable MR	-0.017 (-0.077, 0.044)	0.589				
SBW→Intelligence (n = 2, *F* = 104.07)	IVW	0.261 (0.059, 0.463)	0.011	0.350	NA	NA	NA
	multivariable MR	0.247 (0.157, 0.391)	4.48×10^−6^				

SNP, Single nucleotide polymorphism; MR, Mendelian randomization; PAS, Physical activity status; LST, Leisure screen time; MVPA, Moderate-to-vigorous intensity physical activity during leisure time; SC, Sedentary commuting; IVW, Inverse variance weighted.

**Fig 3:**
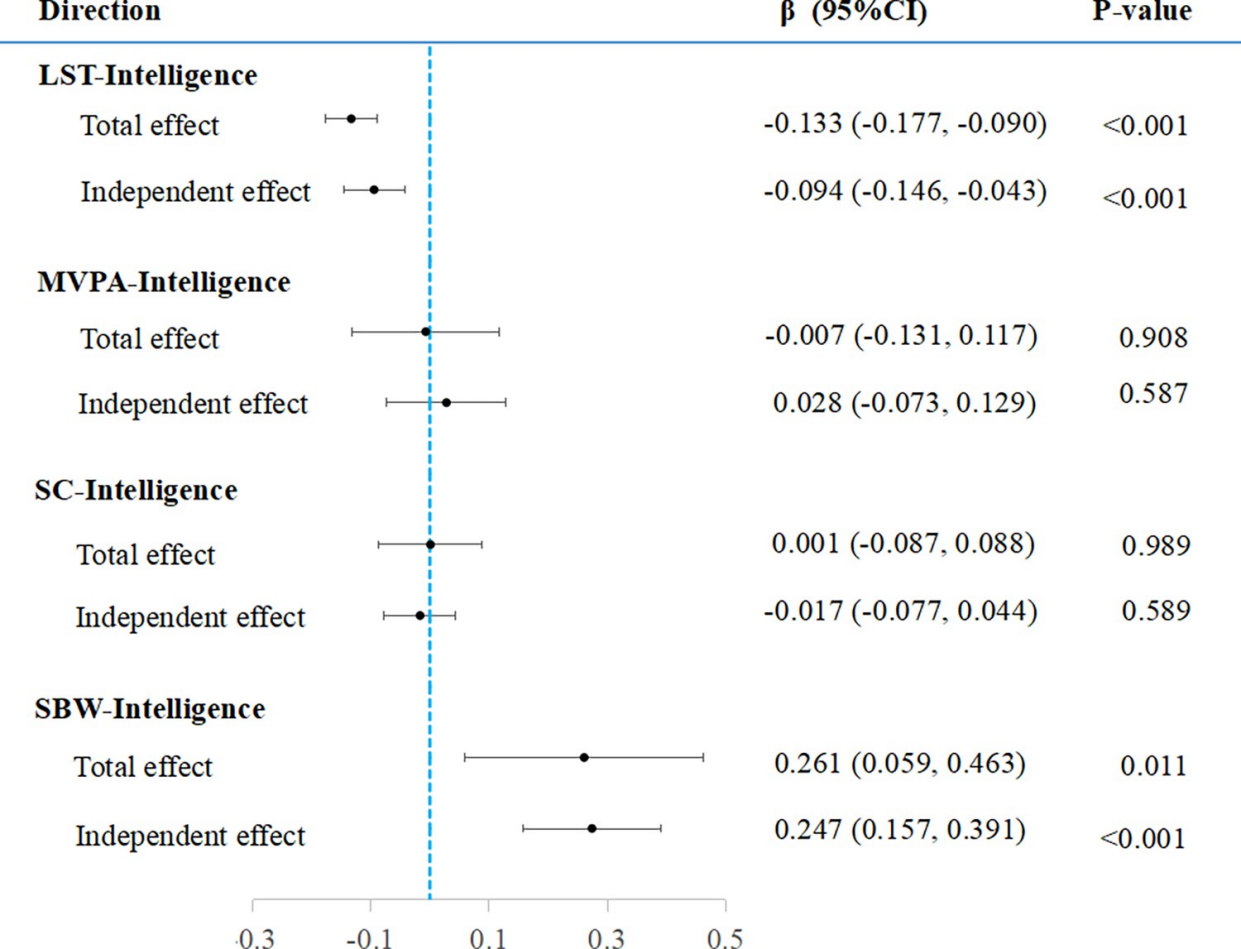
Causal effects for PAS on intelligence. Total effect represents the results of IVW analysis and the independent effect stands for that of multivariable MR analysis. PAS, Physical activity status; LST, Leisure screen time; MVPA, Moderate-to-vigorous intensity physical activity during leisure time; SC, Sedentary commuting; SBW, Sedentary behavior at work; IVW, Inverse variance weighted.

Multivariable MR analysis showed that LST (β = -0.094, 95%CI: -0.146, -0.043, p = 3.35×10^−4^) and SBW (β = 0.274, 95% CI: 0.157, 0.391, p = 4.48×10–6) remained independent influences on intelligence after excluding the interaction between exposure factors (Table 1and Fig 3).

### The causal effect of intelligence on PAS

**Fig 4:**
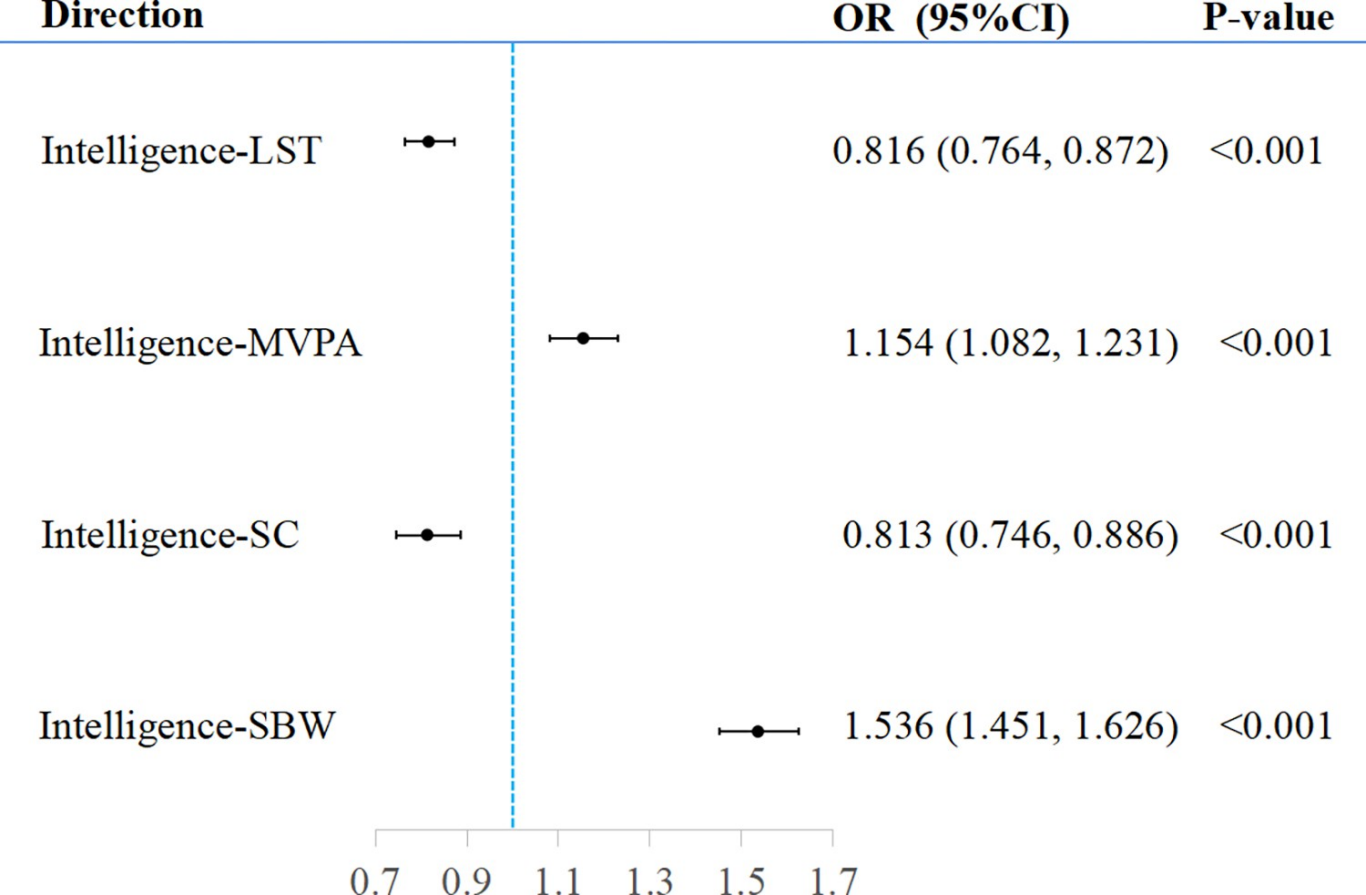
Causal effects for intelligence on PAS. PAS, Physical activity status; LST, Leisure screen time; MVPA, Moderate-to-vigorous intensity physical activity during leisure time; SC, Sedentary commuting; SBW, Sedentary behavior at work; IVW, Inverse variance weighted; OR, Odds ratios.

The results of the reverse MR analysis are shown in Table 2. The findings indicated a significant causal effects of intelligence on PAS, with LST (OR = 0.816, 95%CI: 0.764 to 0.872, *p =* 2.12×10^−9^), MVPA (OR = 1.154, 95%CI: 1.082 to 1.231, *p =* 1.23×10^−5^), SC (OR = 0.813, 95%CI: 0.746 to 0.886, *p =* 2.58× 10^−6^), and SBW (OR = 1.536, 95%CI: 1.451 to 1.626, *p =* 2.00×10^−49^) (Fig 4). MR-Egger, weighted median model with all effects values were in the same direction as IVW. MR-Egger tests showed no pleiotropy for SNPs in each group: LST (Intercept = 0.003, *p =* 0.306), MVPA (Intercept = 0.003, *p =* 0.587), SC (Intercept = 0.004, *p =* 0.390), SBW (Intercept = 0.003, *p =* 0.418) (S5 Fig in S1 File). The leave-one-out method, and plotted funnel plots and forest plots were used to cope with heterogeneity based on the Cochran test, with no individual SNP significantly affected the results (S6-S8 Figs in S1 File). MR-PRESSO test results showed no outliers for SNPs in intelligence to SC and SBW, while SNPs in intelligence to LST and MVPA did not change causality when removing outliers (Table 2 and S12 Table in S2 File). The results of the analysis containing proxy SNPs are seen in agreement with the main results (S14 Table in S2 File).

**Table 2:** Results of Mendelian randomization analysis of intelligence on PAS.

Direction (No.SNPs, *F-statistic*)	Methods	Beta (95%CI)	Beta *P-*value	Cochran’s Q *P*-value	MR-Egger intercept	Intercept *P*-value	MR-PRESSO *P-*value
Intelligence→LST (n = 120, *F* = 35.35)	MR-Egger	0.956 (0.703, 1.300)	0.774	5.71×10^−20^	0.003	0.306	0.563
	IVW	0.816 (0.764, 0.872)	2.12×10^−9^				
	Weighted median	0.842 (0.788, 0.900)	3.86×10^−7^				
Intelligence→MVPA (n = 127, *F* = 35.39)	MR-Egger	1.256 (0.920, 1.714)	0.153	1.48×10^−23^	0.003	0.587	0.457
	IVW	1.154 (1.082, 1.231)	1.23×10^−5^				
	Weighted median	1.141 (1.070, 1.217)	5.87×10^−5^				
Intelligence→SC (n = 127, *F* = 35.39)	MR-Egger	0.965 (0.648, 1.436)	0.859	0.244	0.004	0.390	NA
	IVW	0.813 (0.746, 0.886)	2.58×10^−6^				
	Weighted median	0.825 (0.728, 0.936)	2.71×10^−3^				
Intelligence→SBW (n = 126, *F* = 35.42)	MR-Egger	1.719 (1.303, 2.269)	2.05×10^−4^	0.040	0.003	0.418	NA
	IVW	1.536 (1.451, 1.626)	2.00×10^−49^				
	Weighted median	1.538 (1.425, 1.661)	2.16×10^−28^				

MR-PRESSO test results showed no outliers for SNPs in intelligence on SC and SBW, Distortion Test is not a suitable method. Abbreviation: SNP, Single nucleotide polymorphism; MR, Mendelian randomization; PAS, Physical activity status; LST, Leisure screen time; MVPA, Moderate-to-vigorous intensity physical activity during leisure time; SC, Sedentary commuting; IVW, Inverse variance weighted; OR, Odds ratios.

## Discussion

Our study found that excessive screen exposure during leisure time has a negative effect on our intelligence that is not ameliorated by MVPA, while SBW appears to be beneficial to intelligence. The reverse MR analysis revealed that intelligence has a comprehensive effect on PAS by promoting the performance of moderate-to-vigorous exercise and sedentary behavior during work time, and reducing screen use and sedentary commuting time.

PA is considered as the most cost-effective behavioral intervention for human cognitive function with global concern. Studies suggest that PA might lead to improved cognitive ability in general, and enhanced intelligence scores (IQ) in particular [31, 32]. However, our study were with different findings that those who had MVPA did not show higher levels of intelligence. A systematic review concluded that PA did not show significant benefits in terms of improve intelligence and academic performance [33]. A retrospective cohort study also showed that although there was an association between PA and intelligence, there was no evidence that increasing PA levels might lead to higher intelligence scores [34]. Indeed, the relationship between PA and intelligence is complex, findings have been inconsistent regarding the association between PA and intelligence [33]. Therefore, further randomized controlled trials are needed to elucidate the correlation between PA and brain outcomes, although our findings suggest no significant effect of MVPA on intelligence.

We concur with the majority of studies that excessive screen exposure during leisure time can lead to a reduction in intelligence levels. A prospective cohort study also found a negative association between duration of electronic exposure and IQ development in children from 6 to 72 months [35], which is supported by another study among preterm children [36]. In conclusion, excessive screen exposure can reduce intelligence levels and avoid overuse despite those electronic devices assist our lives and learning.

In this study, SB included SC and SBW. SC has no causal effect on intelligence, however, SBW appears to facilitate intelligence. Falck et al. [37] reported that SB was associated with poorer cognitive performance, yet failed to observe a significant association between the sedentary duration and the incidence of all-cause dementia. In a systematic review of intellectual disability, the authors did not find a causal relationship between sedentary behavior and cognition [38]. More interestingly, Bakrania’s study found that the behavioral content accompanying SB was more closely related to the cognitive level of older adults. For example, if they are watching TV during sedentary behavior, SB was positively associated with cognitive decline; however, if SB occurred in front of a computer for handling problems, then SB was in a negative association with declined cognition [39], and another study obtained similar results [40]. A study from the Cohort Studies of Memory in an International Consortium (COSMIC) also confirmed that specific types of SB may have different effects on cognition [41]. This is consistent with the results of our study.

Regarding the mechanisms underlying the effects of different PAS on intelligence, we speculate that there may be several aspects: 1) Brain structure remodeling: Research indicates that a sedentary lifestyle has deleterious effects on brain structure, which in turn negatively impacts intelligence [42]. Conversely, physical activity has been shown to modify hippocampal volume and subsequently enhance cognitive performance [43]. 2) Brain-derived neurotrophic factor (BDNF) secretion. Previous literature suggests that can play a role in synaptic plasticity and development, neuronal transmission and angiogenesis [44, 45]. 3) Inflammatory factors. Exercise can reduce inflammation and stimulate the release of growth factors and thus brain cell health and function [44, 46].

In the reverse causality study, intelligence seems to influence each PAS. Our results suggest that people with higher level of intelligence seem to prefer exercise than screen exposure. Most previous studies tended to explore the causal effect of PA on intelligence, due to fact that observational studies may bring about reversed causality, the causality of intelligence on PA cannot be ignored. Although we have not seen reports of higher intelligence promoting PA, a decrease in PA participation does exist among those with intellectual disabilities. In a study of intellectually disabled Australian adults, it was found that they participated in sports and PA at significantly lower rates than the general population, which may be somewhat related to their PA impairment [47]. Similarly, significantly lower participation in PA was found in patients with other mental spectrum disorders accompanying intellectual disability [48]. Regarding the effect of intelligence on SB, our results demonstrate that smarter people tend to reduce sedentary commuting while increase sedentary behavior at work, which may be due to the fact that highly intelligent individuals are more likely to get involved in mental rather than physical tasks [34].

Our study explored the bidirectional causal relationship between PAS and intelligence, and drew corresponding conclusions. It is still notable that the causality estimates from MRs are not fully comparable to those from RCTs since MRs represent a lifelong effect rather than that lasts for a certain duration. Furthermore, PA interventions may influence intelligence through other pathways rather than by genetic phenotype exclusively, and vice versa. So far, the causal effect of intelligence on various PAS has not been evaluated in RCTs, possibly due to the ethical and practical limitations.

The main strength of our study is the bidirectional two-sample MR design, which minimizes the influence caused by residual confounding and reverse causality. However, the study also has several limitations. First, the data on PA were derived from self-reports, which may be subjective to some extent. Second, in the case of SC as an exposure factor, we set the significance threshold at *p<*5×10^−6^ due to insufficient IVs and SBW identified only two SNPs as IVs, which may impact the results; third, there is some overlap in the population cohorts for exposure and outcome, and although we calculated no increase in the type I error probability, this may have led to some IVs bias [49] (S15 Table in S2 File).

## Conclusions

In conclusion, our findings suggest that excessive screen exposure is associated with reduced levels of intelligence, while moderate-to-high intensity exercise does not appear to have a significant impact on intelligence. The relationship between sedentary behavior and intelligence is complex, as sedentary work may have a positive effect on intelligence, but further research is needed to confirm this. Additionally, the results of the reverse MR analysis indicate that there is a causal relationship between intelligence and physical activity.

## Supporting information

S1 File (DOCX)

S2 File (XLSX)
