# Supplementary material for: The causality of physical activity status and intelligence: A bidirectional Mendelian randomization study
Source: PLoS One. 2023 Aug 1;18(8):e0289252. doi: 10.1371/journal.pone.0289252 (PMC10393173; doi:10.1371/journal.pone.0289252)
Supplement: S1 File — (DOCX) [file pone.0289252.s001.docx]

**Supplementary Figures** **Content**

**Fig S1.** The scatter plots of the association between genetic predicted physical activity status on intelligence in MR analysis.

**Fig S2.** The leave-one-out analysis of the association between genetic predicted physical activity status on intelligence in MR analysis.

**Fig S3.** The funnel plots of the association between genetic predicted physical activity status on intelligence in MR analysis.

**Fig S4.** The forest plots of the association between genetic predicted physical activity status on intelligence in MR analysis.

**Fig S5.** The scatter plots of the association between of the association between genetic predicted intelligence on physical activity status in MR analysis.

**Fig S6.** The leave-one-out analysis of the association between genetic predicted intelligence on physical activity status in MR analysis.

**Fig S7.** The funnel plots of the association between of the association between genetic predicted intelligence on physical activity status in MR analysis.

**Fig S8.** The forest plots of the association between of the association between genetic predicted intelligence on physical activity status in MR analysis.


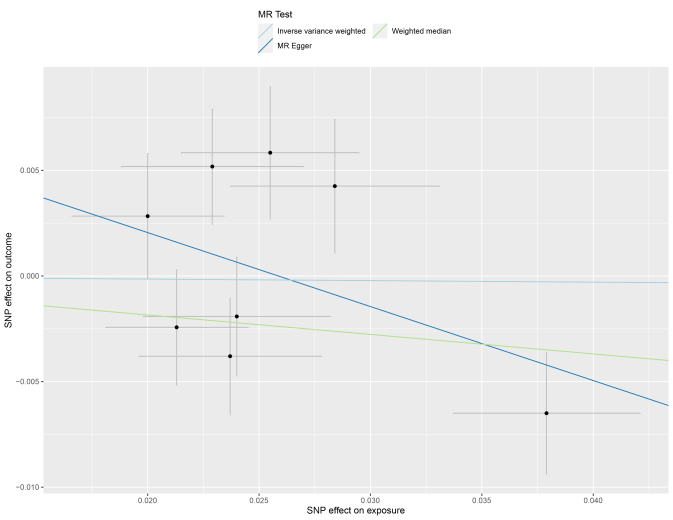

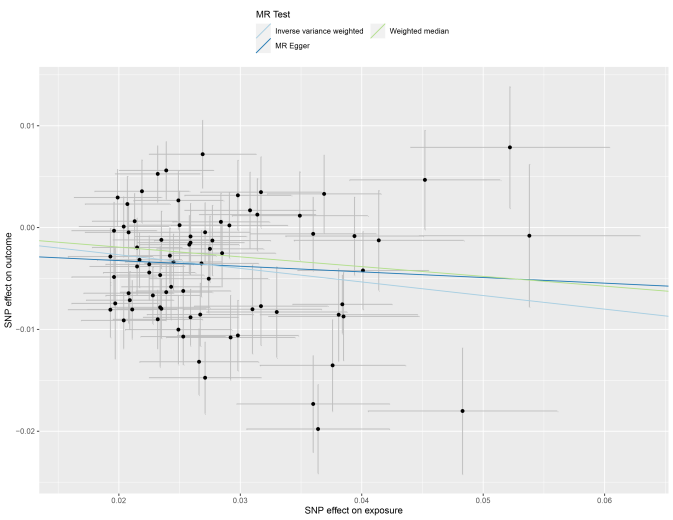


LST on intelligence

MVPA on intelligence


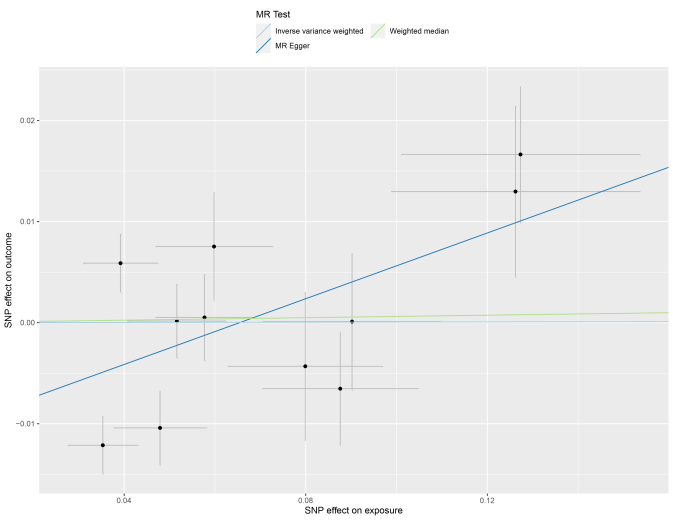

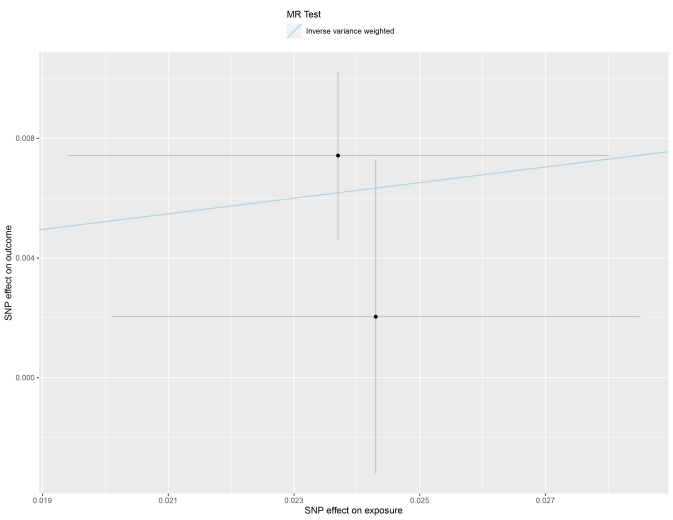


SC on intelligence

SBW on intelligence

**Fig S1.** The scatter plots of the association between genetic predicted physical activity status on intelligence in MR analysis.

MR: Mendelian randomization; LST: Leisure screen time; MVPA: Moderate-to-vigorous intensity physical activity during leisure time; SC: Sedentary commuting; SBW: Sedentary behavior at work.


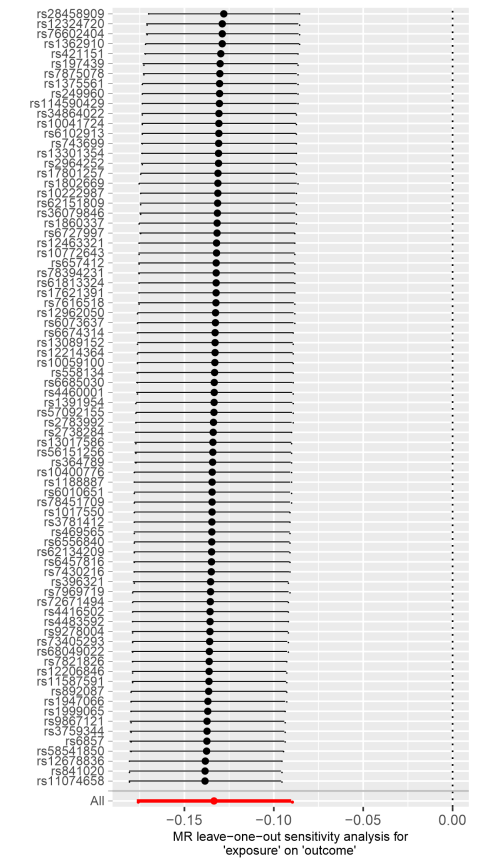

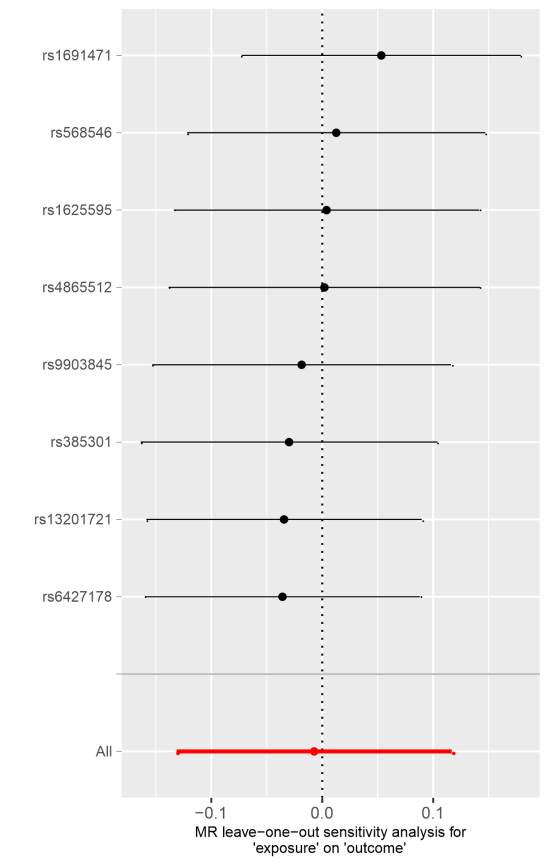

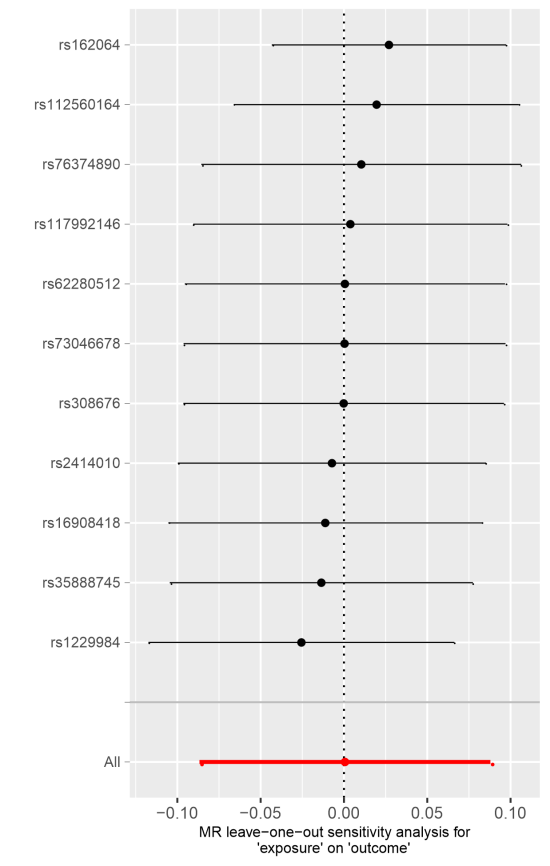


LST on intelligence

MVPA on intelligence

SC on intelligence

10

**Fig S2.** The leave-one-out analysis of the association between genetic predicted physical activity status on intelligence in MR analysis.

MR: Mendelian randomization; LST: Leisure screen time; MVPA: Moderate-to-vigorous intensity physical activity during leisure time; SC: Sedentary commuting.

LST on intelligence

MVPA on intelligence

SC on intelligence

SBW on intelligence


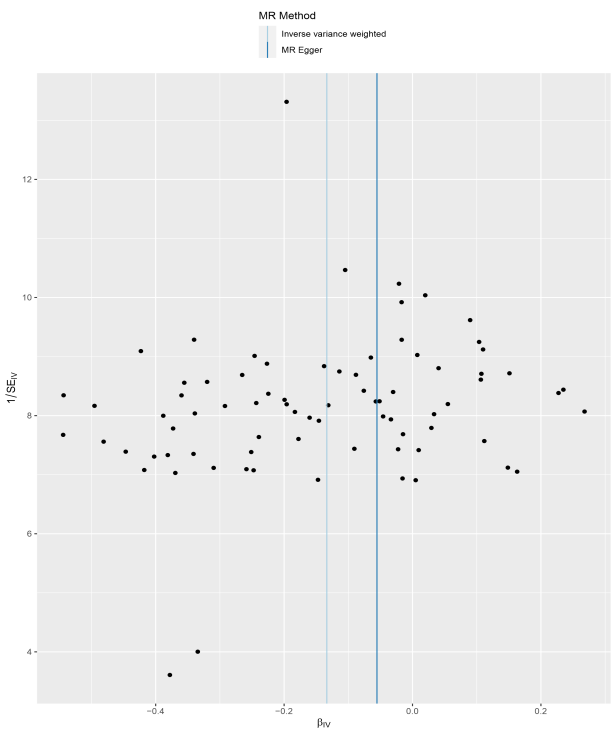

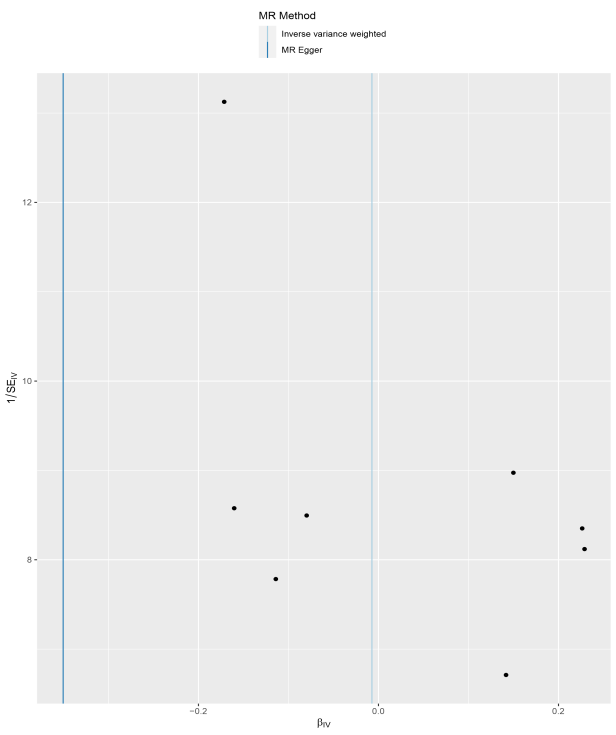

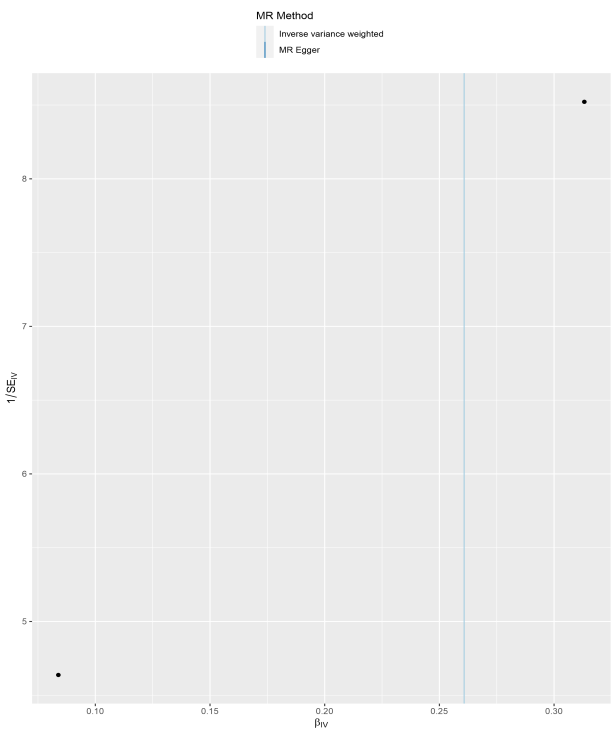

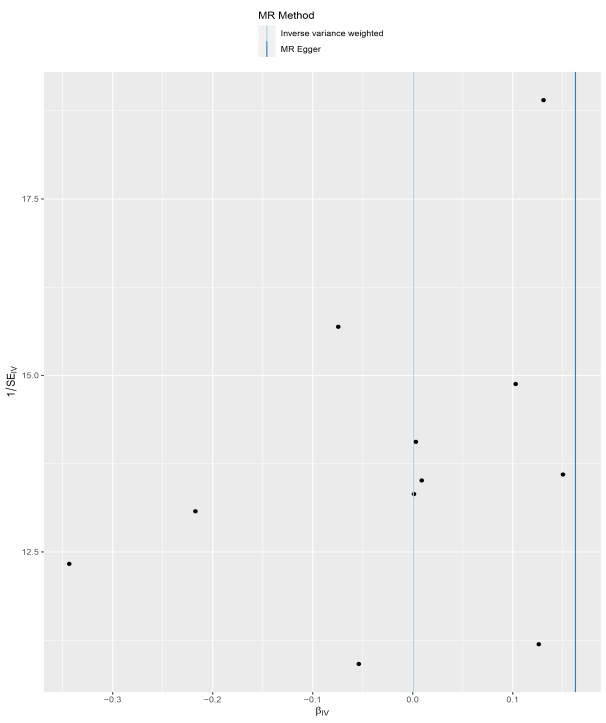


**Fig S3.** The funnel plots of the association between genetic predicted physical activity status on intelligence in MR analysis.

MR: Mendelian randomization; LST: Leisure screen time; MVPA: Moderate-to-vigorous intensity physical activity during leisure time; SC: Sedentary commuting; SBW: Sedentary behavior at work.

SC on intelligence

SBW on intelligence

LST on intelligence

MVPA on intelligence


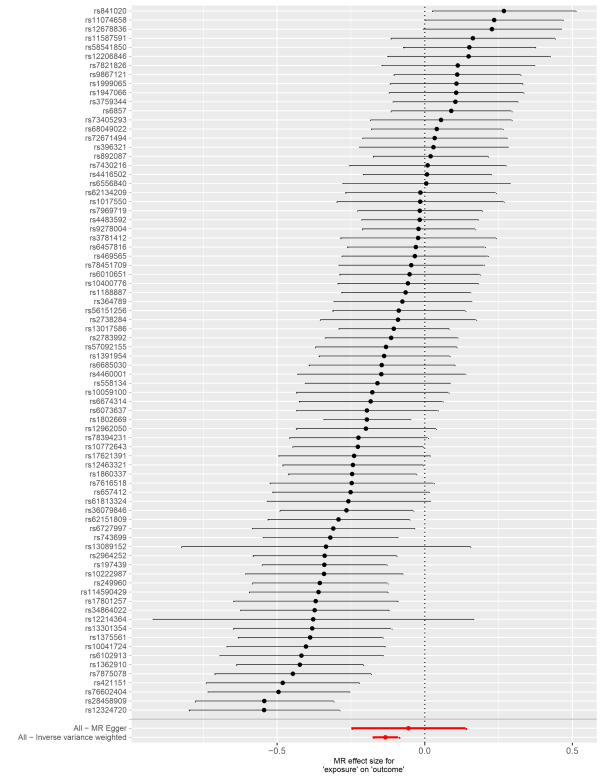

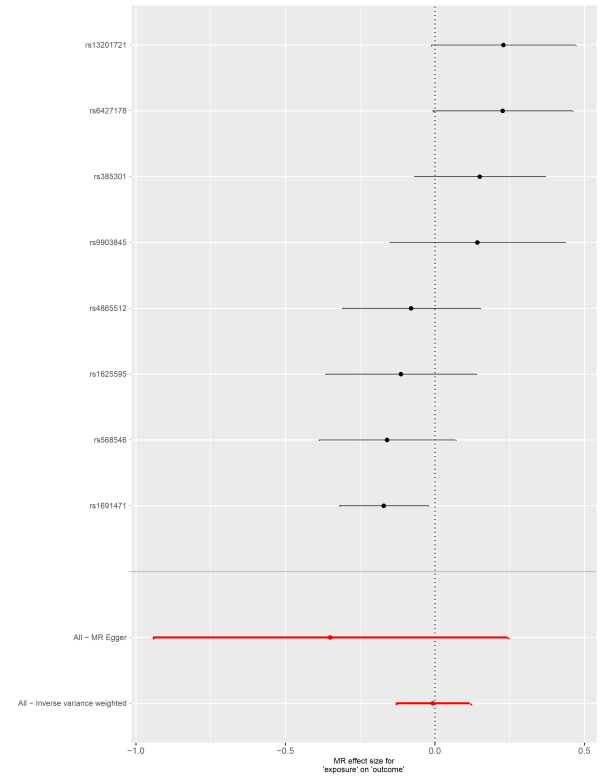

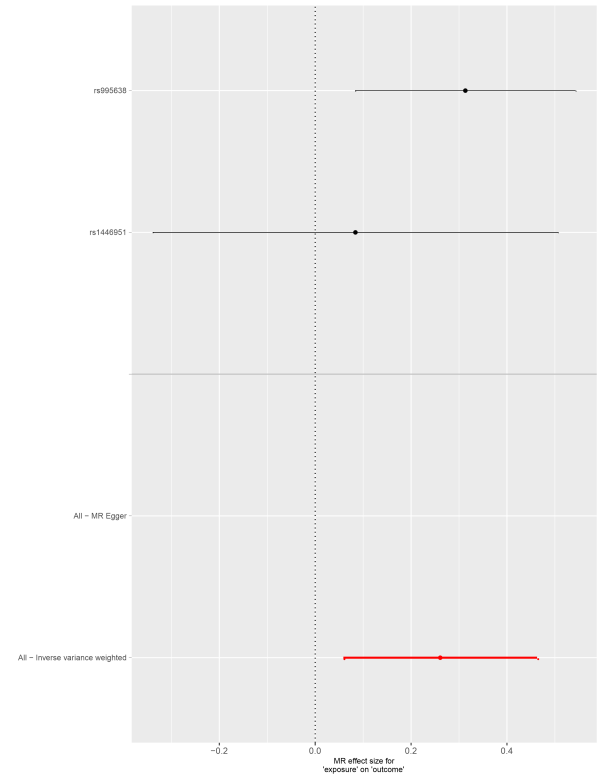

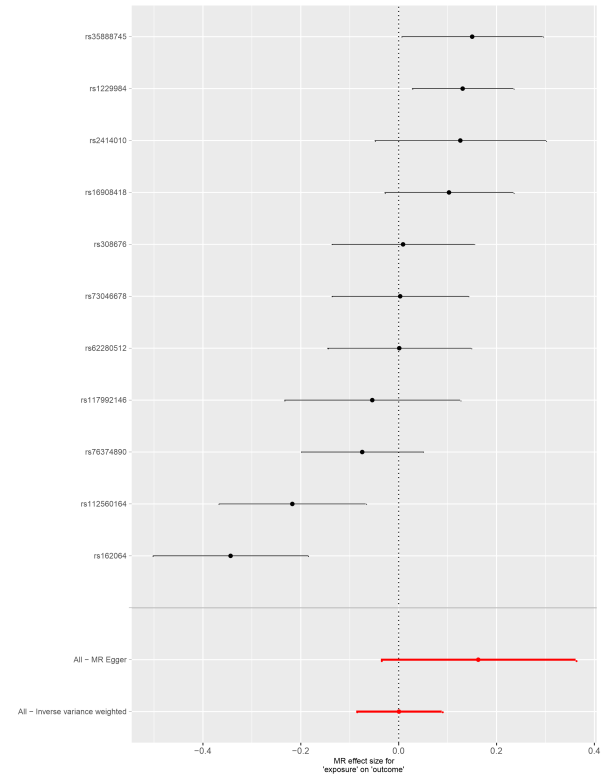


**Fig S4.** The forest plots of the association between genetic predicted physical activity status on intelligence in MR analysis.

MR: Mendelian randomization; LST: Leisure screen time; MVPA: Moderate-to-vigorous intensity physical activity during leisure time; SC: Sedentary commuting; SBW: Sedentary behavior at work.

Intelligence on LST

Intelligence on MVPA

Intelligence on SC

Intelligence on SBW


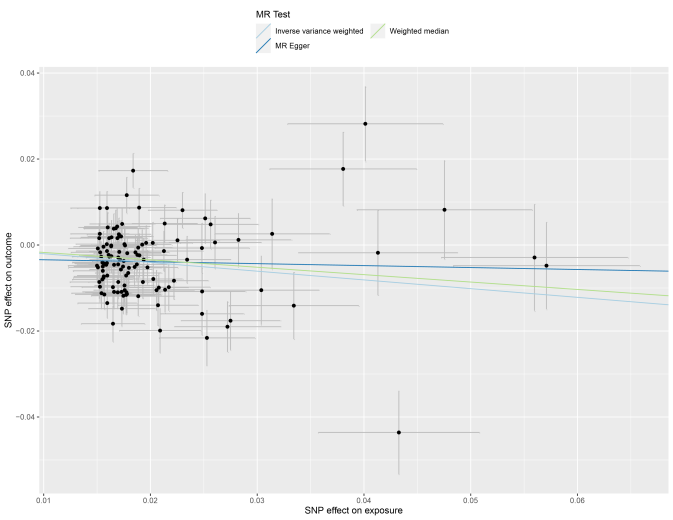

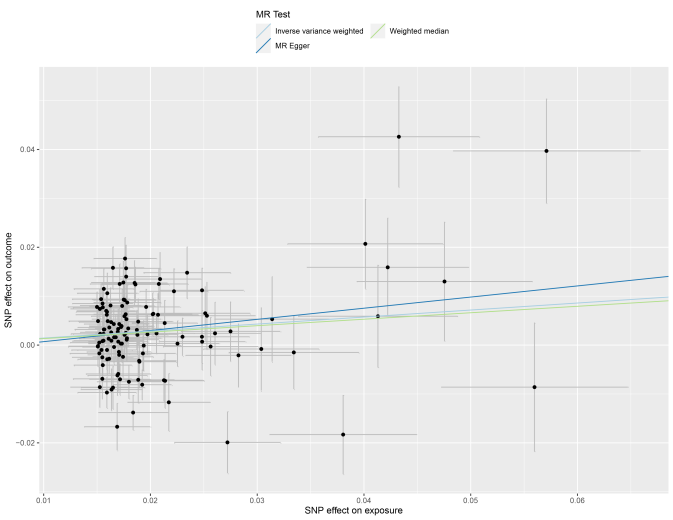

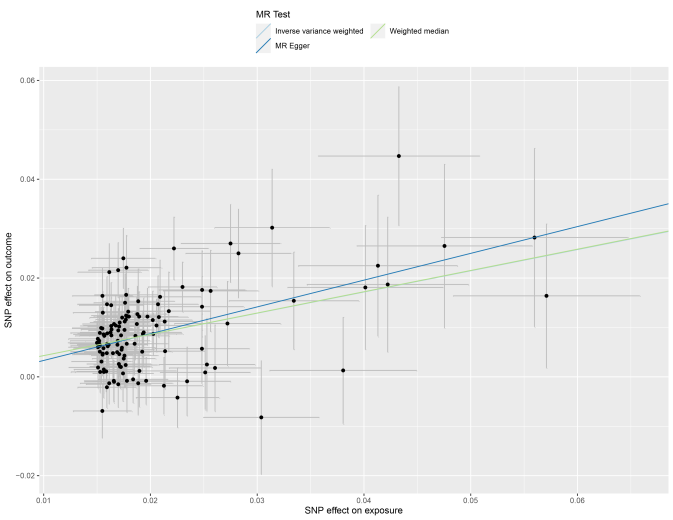

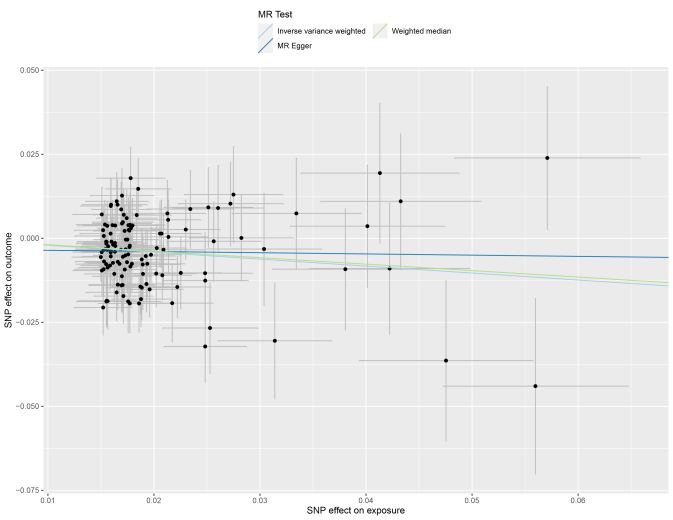


**Fig S5.** The scatter plots of the association between of the association between genetic predicted intelligence on physical activity status in MR analysis.

MR: Mendelian randomization; LST: Leisure screen time; MVPA: Moderate-to-vigorous intensity physical activity during leisure time; SC: Sedentary commuting; SBW: Sedentary behavior at work.


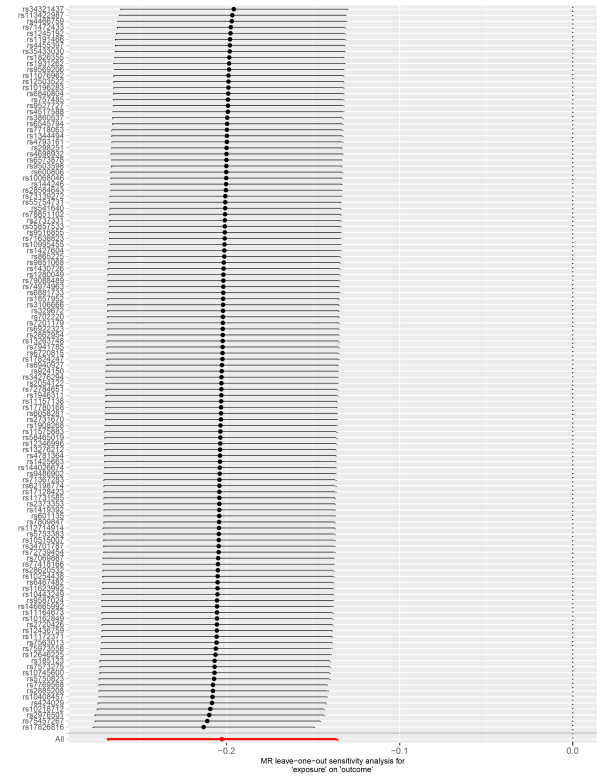

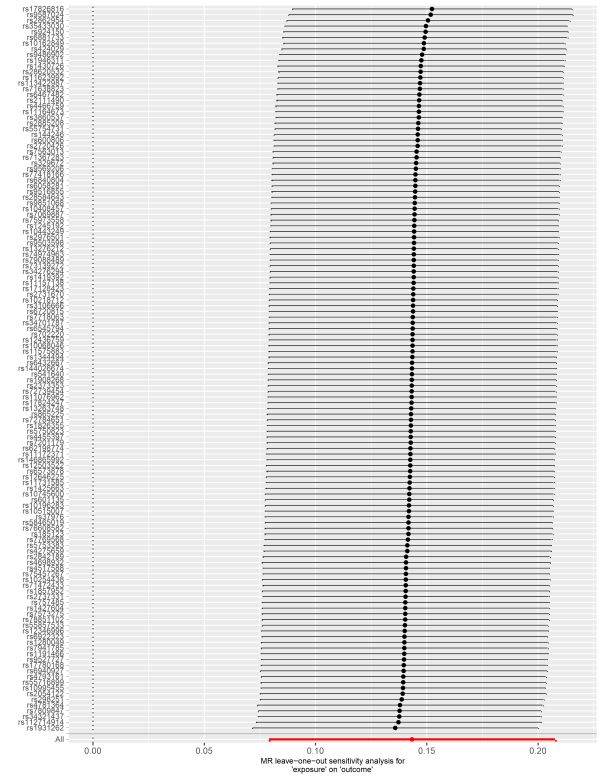


Intelligence on LST

Intelligence on MVPA


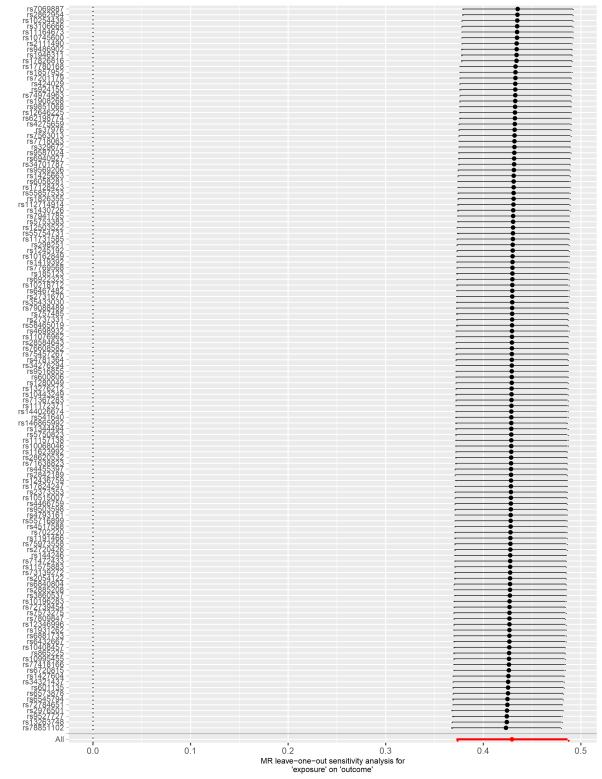

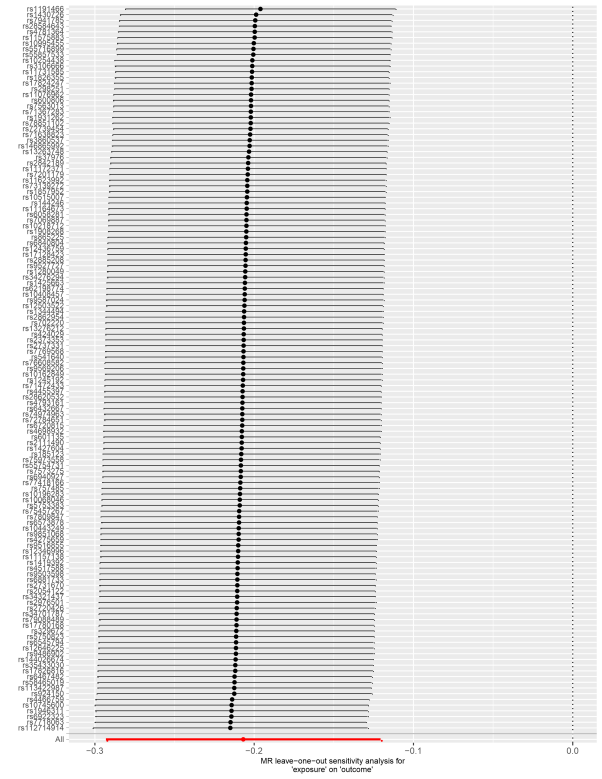


Intelligence on SC

Intelligence on SBW

**Fig S6.** The leave-one-out analysis of the association between genetic predicted intelligence on physical activity status in MR analysis.

MR: Mendelian randomization; LST: Leisure screen time; MVPA: Moderate-to-vigorous intensity physical activity during leisure time; SC: Sedentary commuting; SBW: Sedentary behavior at work.

Intelligence on LST

Intelligence on MVPA

Intelligence on SC

Intelligence on SBW


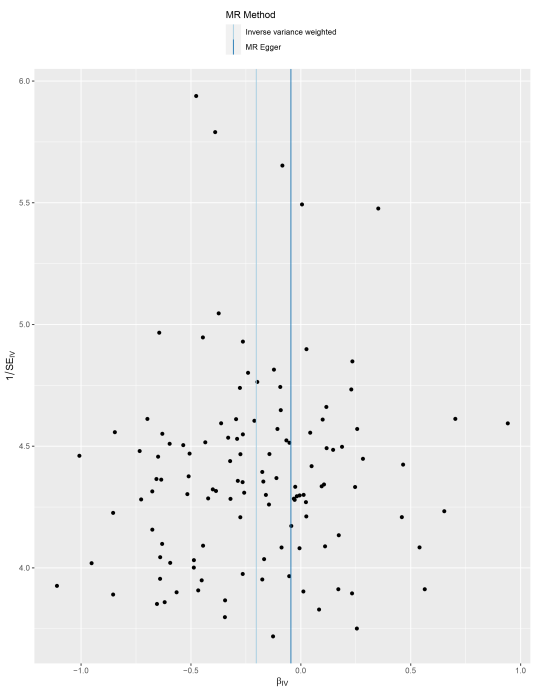

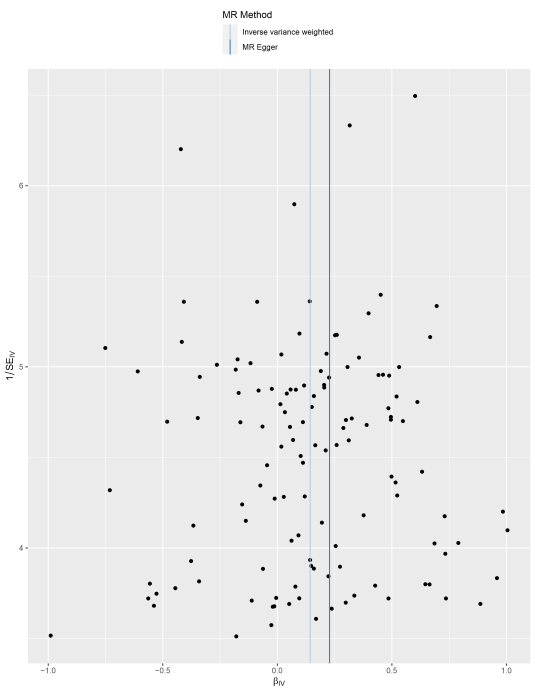

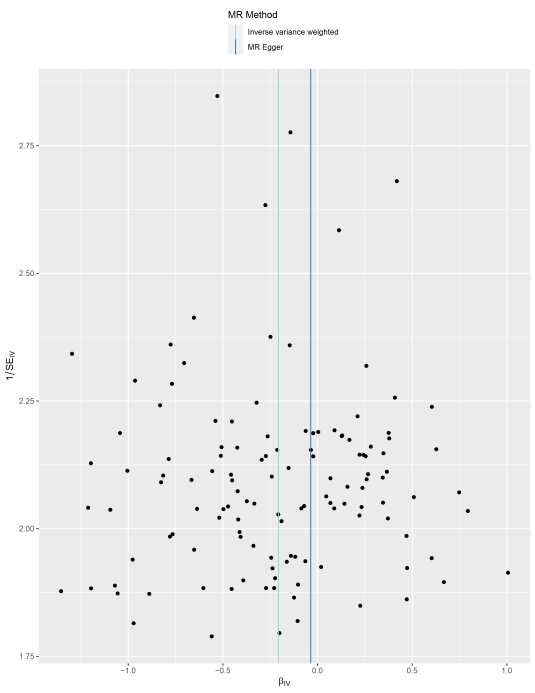

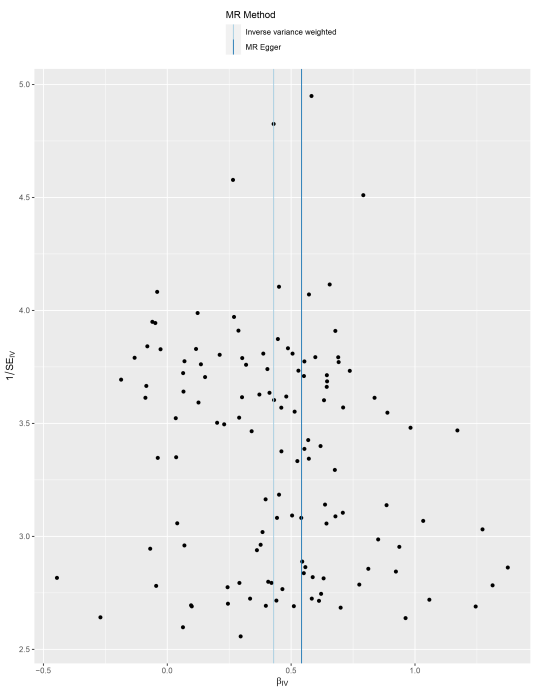


**Fig S7.** The funnel plots of the association between of the association between genetic predicted intelligence on physical activity status in MR analysis.

MR: Mendelian randomization; LST: Leisure screen time; MVPA: Moderate-to-vigorous intensity physical activity during leisure time; SC: Sedentary commuting; SBW: Sedentary behavior at work.

Intelligence on LST

Intelligence on MVPA

Intelligence on SC

Intelligence on SBW


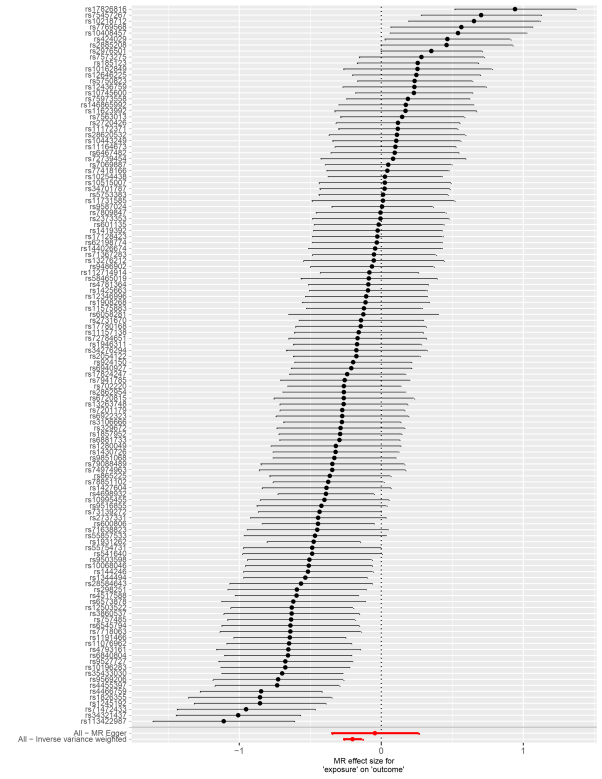

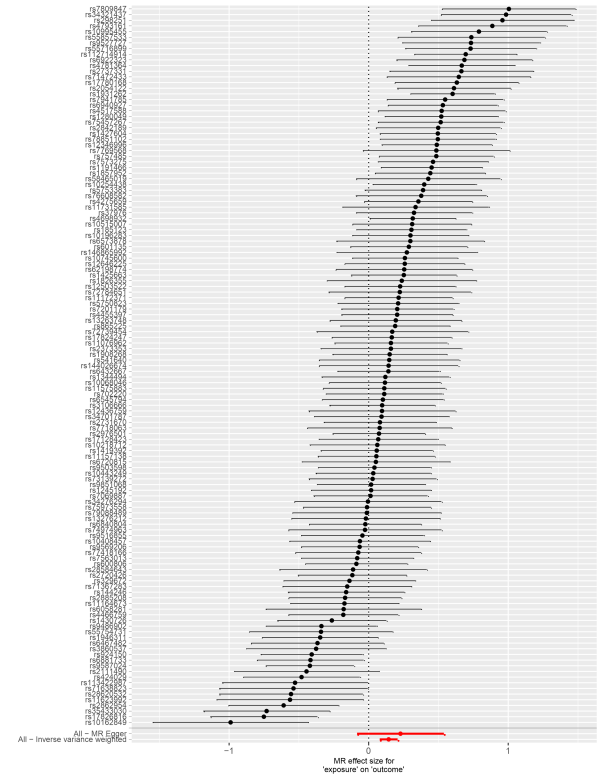

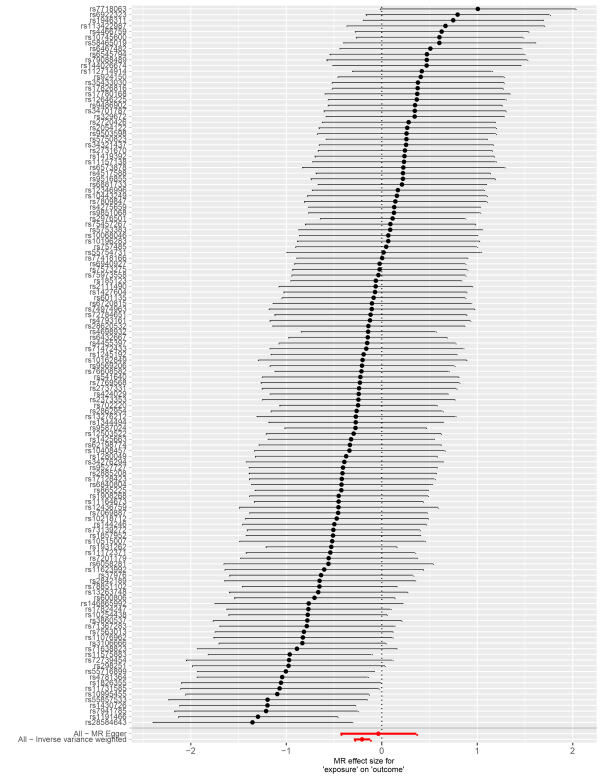

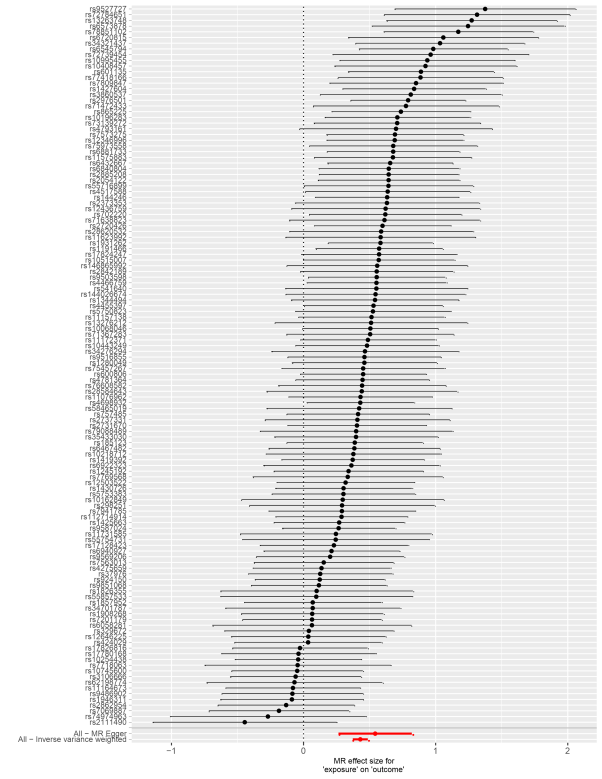


**Fig S8.** The forest plots of the association between of the association between genetic predicted intelligence on physical activity status in MR analysis.

MR: Mendelian randomization; LST: Leisure screen time; MVPA: Moderate-to-vigorous intensity physical activity during leisure time; SC: Sedentary commuting; SBW: Sedentary behavior at work.
